# Supplementary material for: Fast and sensitive flow-injection mass spectrometry metabolomics by analyzing sample-specific ion distributions
Source: Nat Commun. 2020 Jun 24;11:3186. doi: 10.1038/s41467-020-17026-6 (PMC7314751; doi:10.1038/s41467-020-17026-6)
Supplement: Supplementary file 1 — Supplementary Information [file 41467_2020_17026_MOESM1_ESM.pdf]

Supplementary Information

Fast and sensitive flow-injection mass spectrometry metabolomics by analysing sample specific ion distributions

Sarvin et al.

Fast and sensitive flow-injection mass spectrometry metabolomics by analysing sample specific ion distributions

Boris Sarvin,‡[a] Shoval Lagziel,‡[b] Nikita Sarvin,[a] Dzmitry Mukha,[a] Praveen Kumar[a] Elina Aizenshtein[c] and Tomer Shlomi*[abc]

[a] *Faculty of Biology, Technion – Israel Institute of Technology, 32000 Haifa, Israel.*
[b] *Faculty of Computer Science, Technion – Israel Institute of Technology, 32000 Haifa, Israel.*
[c] *Lokey Center for Life Science and Engineering, Technion – Israel Institute of Technology, 32000 Haifa, Israel.*
‡ B.S. and S.L. contributed equally to this work.

Corresponding Author

*E-mail: tomersh@cs.technion.ac.il. Address: Emerson Building, Technion – Israel Institute of Technology, 32000 Haifa, Israel. Phone: +972 077 877 1767

Table of Contents

Supplementary Figure 1 3
Supplementary Figure 2 4
Supplementary Figure 3 5
Supplementary Figure 4 6
Supplementary Figure 5 7
Supplementary Figure 6 8
Supplementary Figure 7 9
Supplementary Figure 8 10
Supplementary Figure 9 11
Supplementary Figure 10 12
Supplementary Figure 11 13
Supplementary Figure 12 14
Supplementary Figure 13 15
Supplementary Figure 14 16
Supplementary Figure 15 17
Supplementary Table 1 18
Supplementary Table 2 19
Supplementary Table 3 20
Supplementary Table 4 21
Supplementary Table 5 22
Supplementary References 23

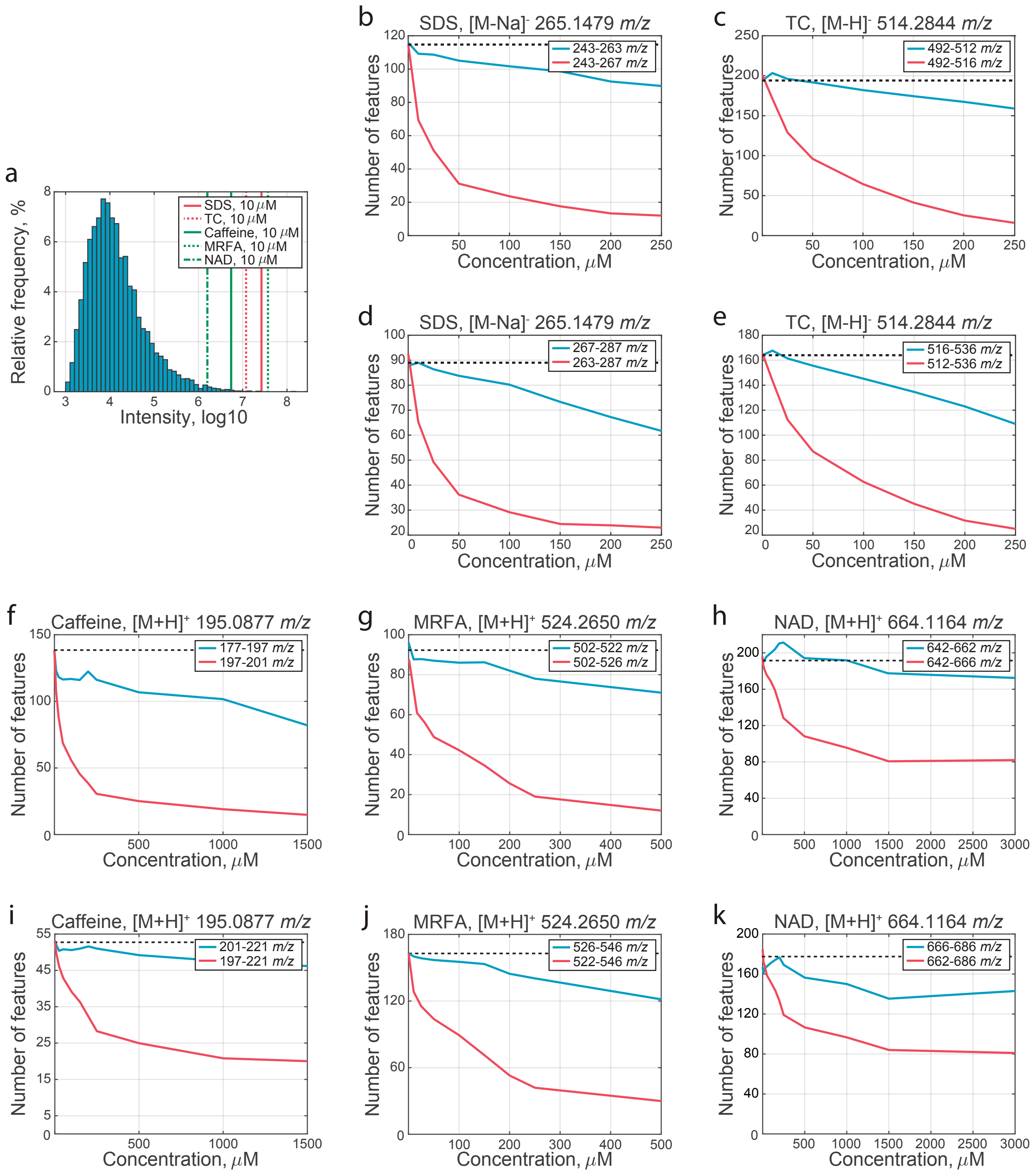

Supplementary Figure 1 Ion competition and ion suppression effects in the metabolomics analysis induced by adding a series of increasing concentrations of five compounds, evaluated via different scan ranges. **a** The distribution of log_{10} intensities of reproducible *m/z* features detected by FI-MS based metabolomics analysis of serum samples with 20 *m/z* scan ranges in negative ionization mode. The measured ion intensity of SDS (sodium dodecyl sulfate; $[M\text{-}H]^-$ – 265.1479 *m/z*; in red), TC (taurocholic acid; $[M\text{-}H]^-$ – 514.2844 *m/z*; in red, dotted line), caffeine ($[M\text{-}H]^+$ – 195.0877 *m/z*; in green), MRFA (Met-Arg-Phe-Ala peptide; $[M\text{-}H]^+$ – 524.2650 *m/z*; in green, dotted line) and NAD (β-nicotinamide adenine dinucleotide; $[M\text{-}H]^+$ – 664.1164 *m/z*; in green, dashed line) when adding a minimal concentration of 10 µM of each compound. **b-k** The number of significant *m/z* features found (within the narrower scan range) when scanning for the 20 *m/z* scan range (in blue) and for the 24 *m/z* scan range (in red), adding increasing concentrations of SDS (**b, d**), TC (**c, e**), Caffeine (**f, i**), MRFA (**g, j**) and NAD (**h, k**); black horizontal line represents the number of *m/z* features detected without adding these compounds. Source data are provided as a Source Data file.

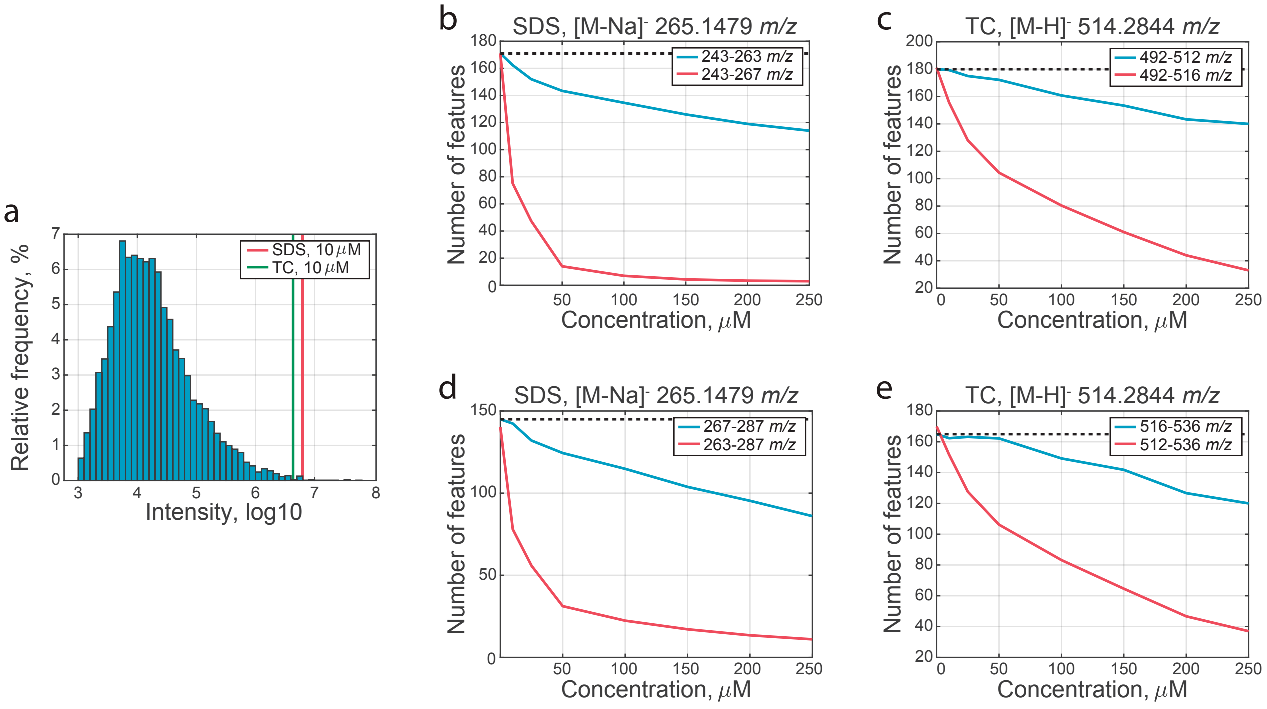

Supplementary Figure 2 Ion competition and ion suppression effects in the lipidomics analysis induced by adding a series of increasing concentrations of two compounds, evaluated via different scan ranges. **a** The distribution of $\log_{10}$ intensities of reproducible *m/z* features detected by FI-MS based lipidomics analysis of serum samples with 20 *m/z* scan ranges in negative ionization mode. The measured intensity of SDS (sodium dodecyl sulfate; $[M\text{-}H]^-$ – 265.1479 *m/z*; in red) and of TC (taurocholic acid; $[M\text{-}H]^-$ – 514.2844 *m/z*; in green), when adding a minimal concentration of 10 µM of SDS and TC. **b-e** The number of significant *m/z* features found (within the narrower scan range) when scanning for the 20 *m/z* scan range (in blue) and for the 24 *m/z* scan range (in red), adding increasing concentrations of SDS (**b, d**) and TC (**d, e**); black horizontal line represents the number of *m/z* features detected without adding SDS/TC. Source data are provided as a Source Data file.

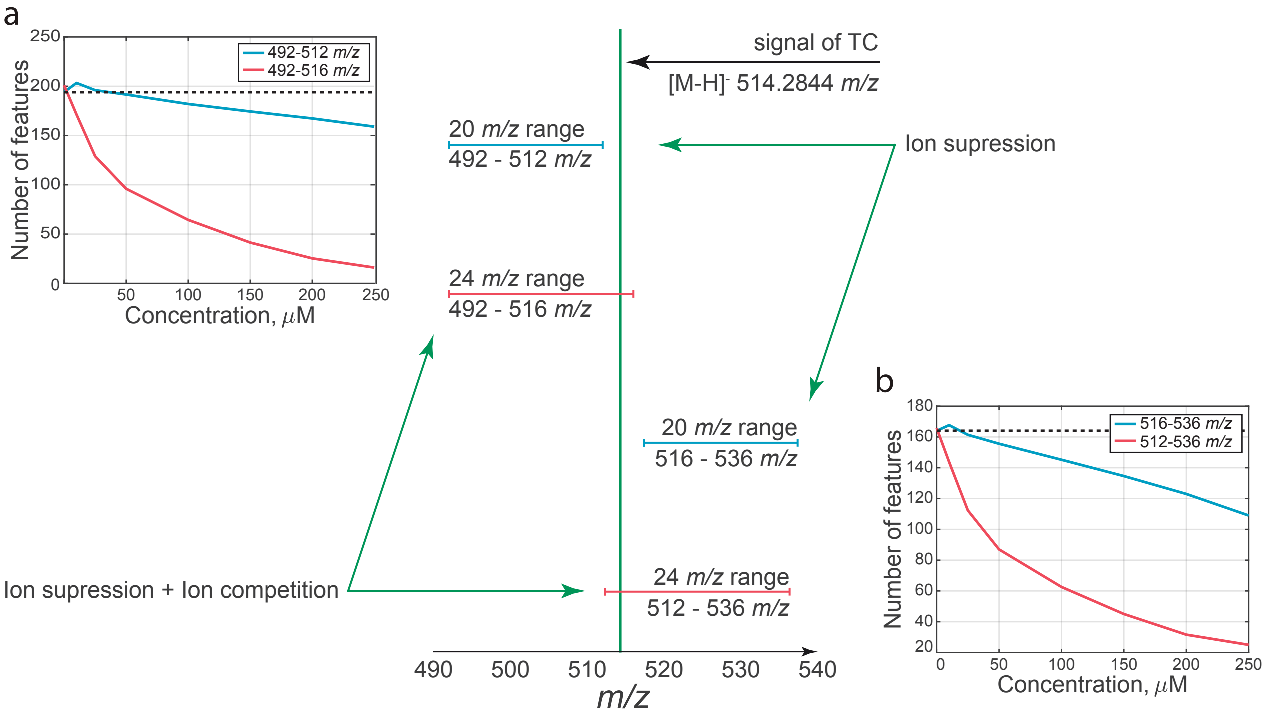

Supplementary Figure 3 An experimental scheme for investigating ion suppression and ion competition effects in FI-MS analysis: Gradually inducing the ion flow by adding increasing concentrations of some compound to the analyte, while configuring the mass spectrometer to scan for two overlapping ranges that include or exclude the added compound; here, taurocholic acid (TC; $[M-H]^-$ – 514.2844 *m/z*) was added to metabolite extracts from serum samples, while a 20 *m/z* scan range, which excludes this compound and an overlapping 24 *m/z* scan range that includes it are scanned. We repeat the experiment twice: Once, limiting the mass-spec scan window to 20 *m/z* and 24 *m/z* ranges that start at the ion of added compound *m/z* minus 22 *m/z* (at *m/z* of 492); and second, in which the 20 *m/z* and 24 *m/z* ranges end at the ion of added compound *m/z* plus 22 Da (at *m/z* of 536). **a, b** The number of significant *m/z* features found (within the narrower scan range; y-axis) when scanning for the 20 *m/z* scan range (in blue) and for the 24 *m/z* scan range (in red), adding increasing concentrations of TC (x-axis).

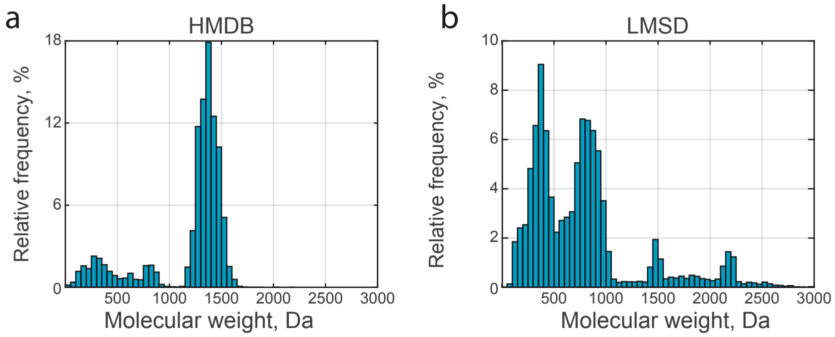

Supplementary Figure 4 Molecular weight distributions of metabolites from the Human Metabolome Database[1] (HMDB; **a**) and of lipids from the LIPID MAPS Structure Database[2] (LMSD; **b**).

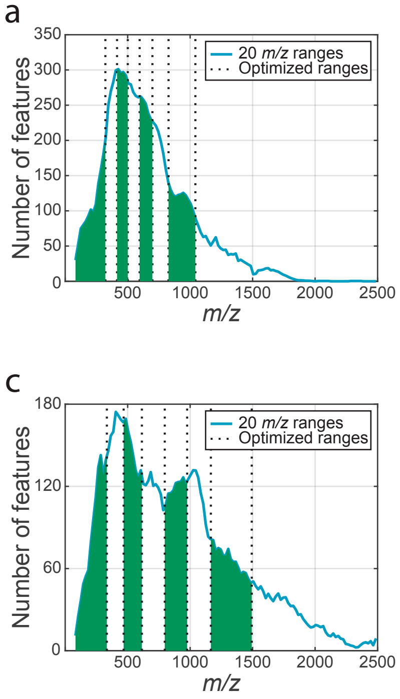

Supplementary Figure 5 The optimized 8 scan ranges determined for FI-MS based metabolomics analysis of serum samples in negative (**a**) and positive (**b**) ionization modes, and for lipidomics analysis in negative (**c**) and positive (**d**) ionization modes. Source data are provided as a Source Data file.

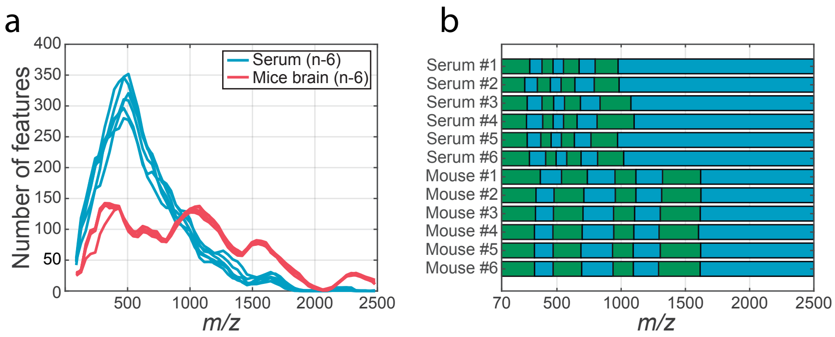

Supplementary Figure 6 Reproducibility of the distribution of *m/z* features for different samples of the same type. **a** Distribution of reproducible *m/z* features based on 64 ranges exhaustive spectral-stitching experiment (see Methods) for 6 different serum samples of healthy individuals (in blue) and 6 extracts of brain tissue of mice (in red). **b** Sets of optimized ranges for FI-MS with 8 ranges in negative ionization mode based on the obtained reproducible *m/z* features distributions. Source data are provided as a Source Data file.

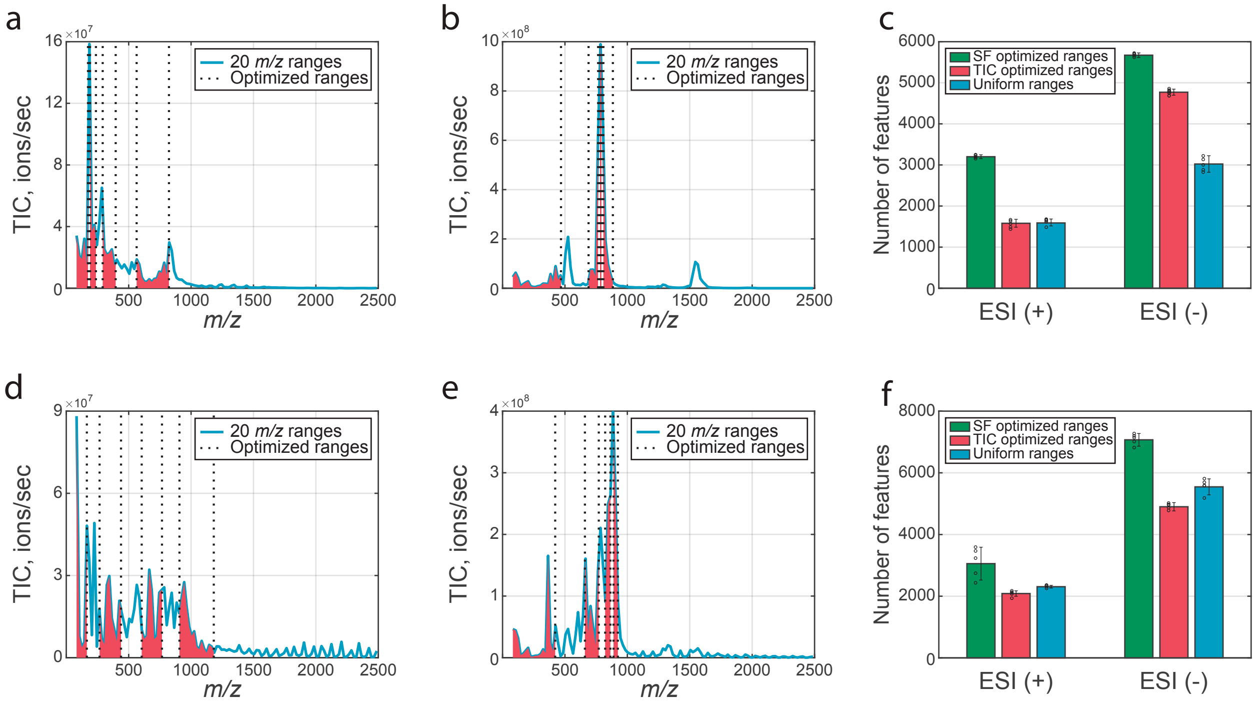

Supplementary Figure 7 Optimizing the sensitivity of rapid flow injection mass spectrometry based on total ion count (TIC) distribution. **a-b, d-e** Optimized sets of 8 scan ranges calculated based on the TIC distribution for metabolomics analysis of samples in negative (**a**) and positive (**b**) ionization modes; and for lipidomics analysis in negative (**d**) and positive (**e**) ionization modes. **c, f** The number of reproducible *m/z* features identified with FI-MS method using the latter TIC optimized scan ranges (in red), using the optimized sets of ranges based on the distribution of the number of reproducible *m/z* features (in green), and using uniform ranges (in blue) for metabolomics (**c**) and lipidomics (**f**) analysis. Data are mean ± SD, n = 5 independent repetitions of the FI-MS analysis. Source data are provided as a Source Data file.

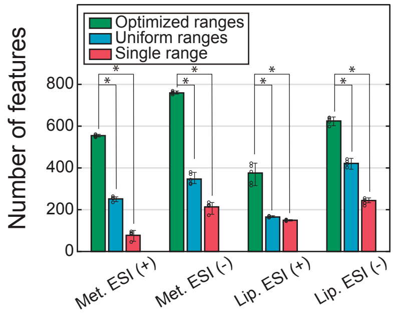

Supplementary Figure 8 The number of putatively annotated *m/z* features for measurements performed with our optimized ranges FI-MS method (green), uniform ranges FI-MS (blue), and single range FI-MS (red). *$P < 10^{-7}$ by two-sample t-test. Data are mean ± SD, n = 5 independent repetitions of the FI-MS analysis. Source data are provided as a Source Data file.

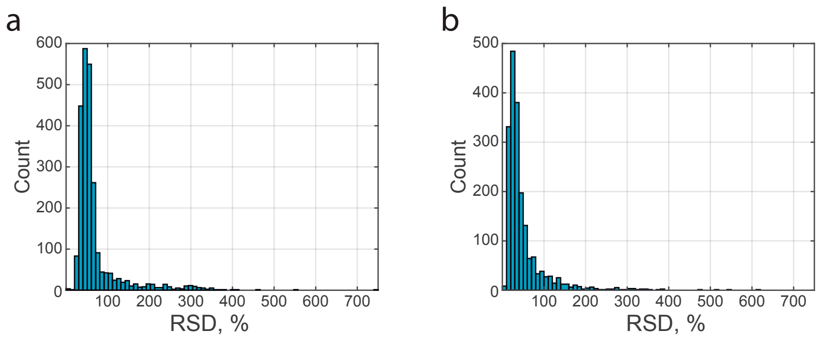

Supplementary Figure 9 Distribution of RSD values for reproducible *m/z* features measured across 98 serum samples of healthy individuals in negative (**a**) and positive (**b**) ionization modes. Source data are provided as a Source Data file.

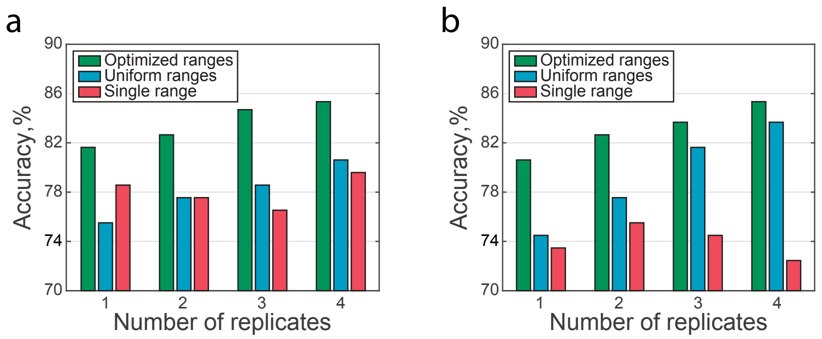

Supplementary Figure 10 Accuracy of gender prediction across 98 serum samples of healthy individuals based on measurements performed with our optimized ranges FI-MS method (green), uniform ranges (blue), and using a single range (red), considering 1, 2, 3, and 4, repeated injections of each sample, in negative (**a**) and positive (**b**) ionization modes. Considering a single injection of each serum sample, the measured intensities provided a gender prediction accuracy of 82% and 80%, in negative and positive ionization modes; comparable to those reported with the LC-MS analysis[3] (85% and 78%, in negative and positive modes, respectively). A lower accuracy of 75% and 74% was obtained with the uniform-range FI-MS; and 78% and 74% with a single-range FI-MS. The improved accuracy obtained with our optimized ranges FI-MS method is further observed when performing multiple injections of each sample to lower noise and considering the median intensity per sample. Source data are provided as a Source Data file.

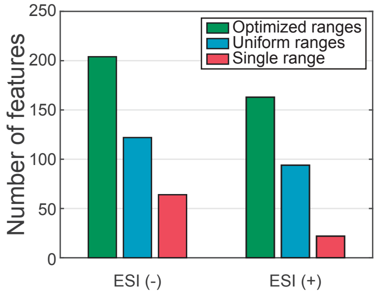

Supplementary Figure 11 Number of *m/z* features (y-axis) detected by our optimized ranges FI-MS method (green), uniform range (spectral-stitching) FI-MS (blue), and single range FI-MS (red) across a panel of 10 cell lines (HeLa, Hek293, HepG2, MiaPaca2, HCT116, Panc-1, A549, WM266-4, Jurkat and CCRF-CEM) whose intensity profile across cell lines is significantly correlated with corresponding LC-MS measurements (FDR corrected Pearson $p < 0.05$), in negative and positive modes. Source data are provided as a Source Data file.

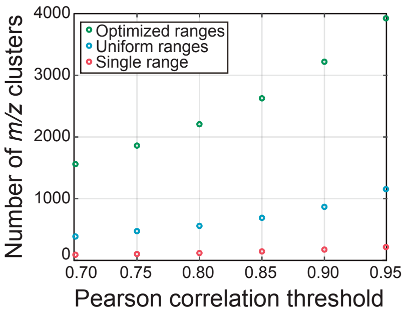

Supplementary Figure 12 The number of *m/z* clusters identified with metabolomics analysis of 98 serum samples (y-axis), performed with our optimized ranges FI-MS method (green), uniform ranges FI-MS (blue), and single range FI-MS (red); considering a range of thresholds on the minimal correlation between the measured intensity of *m/z* features across samples that are clustered together (x-axis). We repeated the clustering considering a range of thresholds for the minimal correlation between m/z features that are grouped together (from 0.7 to 0.95), finding a markedly higher number of m/z clusters for our optimized ranges FI-MS method versus for uniform ranges FI-MS and single range FI-MS for all thresholds tested. Source data are provided as a Source Data file.

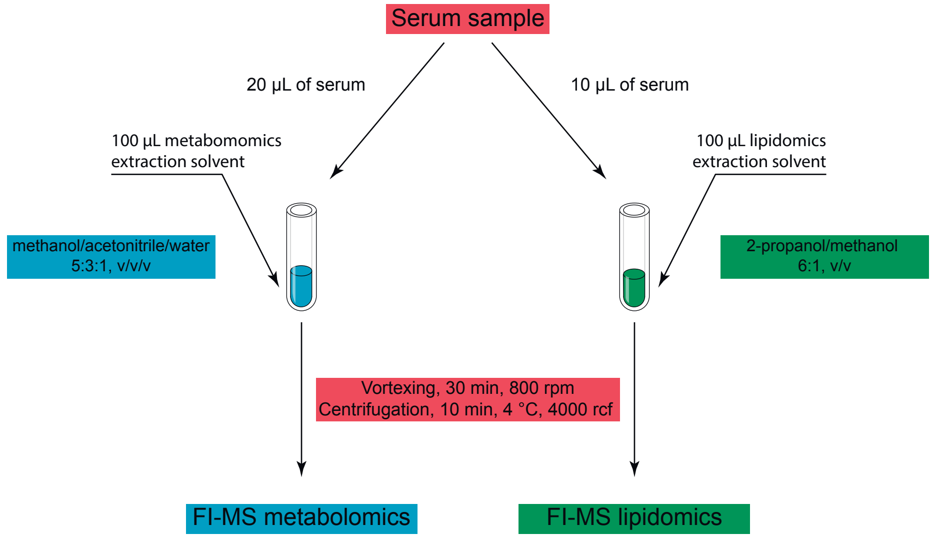

Supplementary Figure 13 Extraction protocols of serum samples for metabolomics and lipidomics FI-MS analysis.

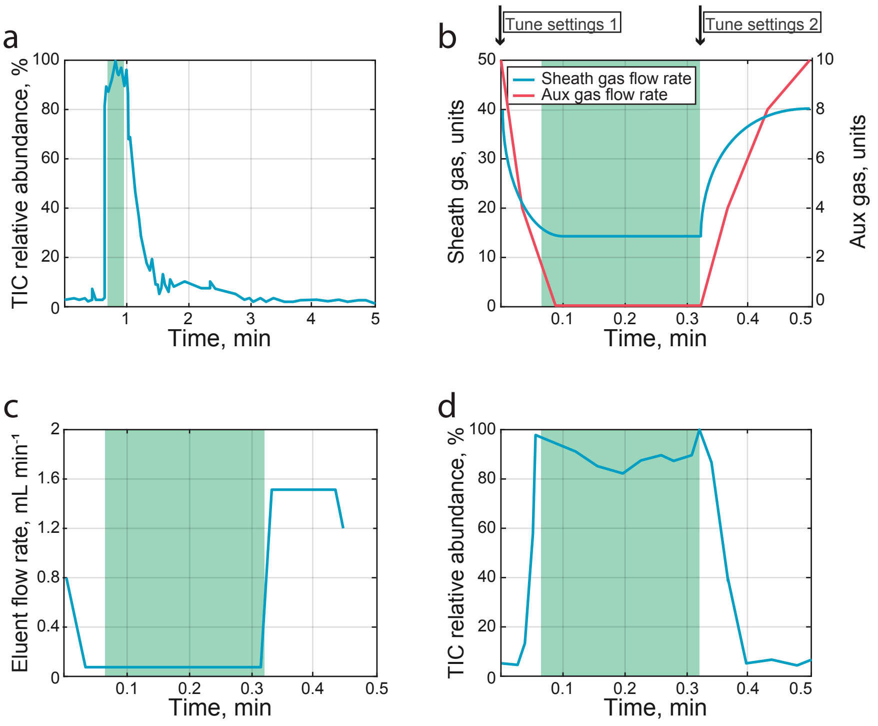

Supplementary Figure 14 Optimization of flow rate gradient for FI-MS analysis. **a** TIC chromatogram of FI-MS serum sample analysis in isocratic mode; high and stable TIC is obtained within a 0.3 min interval (in green). **b** Schematic representation of sheath (in blue) and aux (in red) gases flow dynamics achieved by switching between two mass spectrometer tune files; 1st on time zero, switching to low gases flow rates that stabilizes before the beginning of scanning (where eluent flow is low; in green), and 2nd, on time 0.32 min, switching to high gases flow rates for humidity control during a washing step (where eluent flow is high). **c** An optimal gradient of eluent flow minimizing the total injection cycle time (with 75 µL min^{-1} flow rate during scanning; in green). **d** TIC chromatogram of FI-MS serum sample analysis with the optimal gradient elution flow; high and stable TIC is obtained within a 0.25 min interval (in green). Source data are provided as a Source Data file.

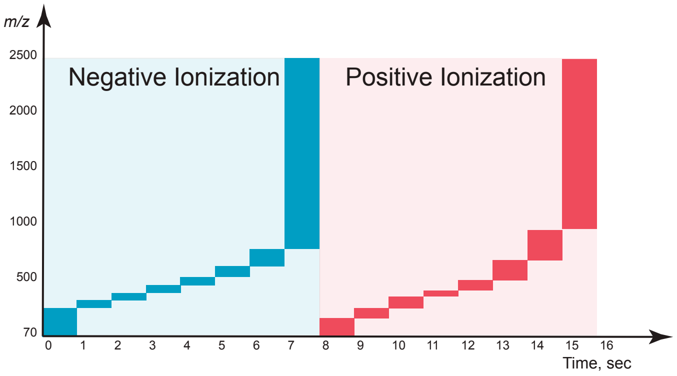

Supplementary Figure 15 The configured scan ranges (y-axis) and ionization modes (negative in blue; and positive in red) throughout the ~15 seconds data acquisition period (x axis) in our FI-MS method.

Supplementary Table 1 Quantitative performance of the optimized ranges FI-MS method for metabolites and lipids

Compound	Ion type	Limit of detection, nM	Linear dynamic range, nM	Correlation coefficient, R^2
Arginine	$[M+H]^+$	20	40 – 8000	0.9975
Glucose 6-phosphate	$[M-H]^-$	30	50 – 6000	0.9972
MRFA	$[M+H]^+$	20	40 – 6000	0.9899
15:0-18:1 (d7) phosphocholine	$[M-H]^-$	<150	150 – 12000	0.9966
15:0-18:1 (d7) phosphoethanolamine	$[M-H]^-$	<20	20 – >450	0.9963
15:0-18:1 (d7) phospho-L-serine	$[M-H]^-$	40	80 – >300	0.9844
15:0-18:1 (d7) phosphoglycerol	$[M-H]^-$	<150	150 – >2200	0.9965
15:0-18:1 (d7) phosphoinositol	$[M-H]^-$	45	75 – >600	0.9918
15:0-18:1 (d7) glycerophosphate	$[M-H]^-$	30	45 – >600	0.9933
18:1 (d7) phosphoethanolamine	$[M+H]^+$	30	45 – >600	0.9924

Supplementary Table 2a The concentrations of chemical standards used for quantifying the absolute concentration of serum amino based on the standard addition method

Compound	Concentration, µM		
	1st addition	2nd addition	3rd addition
alanine	20.41	61.24	122.47
aspartic acid	17.29	51.88	103.76
phenylalanine	20.40	61.21	122.42
proline	22.61	67.83	135.65
tryptophan	16.26	48.78	97.55
tyrosine	16.85	50.55	101.11
valine	20.51	61.54	123.08
asparagine	16.78	50.33	100.67
lysine	22.13	66.39	132.79

Supplementary Table 2b The concentration of isotopic chemical standards used for quantifying the absolute concentration of serum lipids based on internal calibration curves

Relative concentration	Compound / Concentration, nM	
	18:1 (d7) lyso phosphoethanolamine	18:1 (d7) lyso phosphocholine
1	50.5	225
2.5	126	563
5	252	1127
10	505	2250
15	760	3380
20	1010	4510

Supplementary Table 3 Differences in FI-MS based measured intensity of intracellular metabolites in HCT116 cancer cells grown in hypoxia versus normoxia (see Methods); focusing on compounds previously shown to have increased concentration in hypoxia based on LC-MS analysis[4]. The column p_FDR shows the FDR corrected p-value for multiple hypothesis testing.

Compound	*m/z*	Ionization mode	p_FDR	p-Value	Fold change Hypoxia Vs Normoxia
GPC	258.1102	POS	0.00203	0.00013	6.34
L-Proline	116.0707	POS	0.00203	0.00010	6.15
Glycerolphosphate	171.0064	NEG	0.00361	0.00058	7.38
Lyso PC 524	524.3709	POS	0.00361	0.00055	2.86
Lyso PC 549	496.3396	POS	0.00361	0.00052	2.67
Glycerylphosphorylethanolamine	214.0487	NEG	0.00701	0.00158	6.16
NAAG	303.0834	NEG	0.00701	0.00136	2.83
L-Asparagine	131.0462	NEG	0.01004	0.00324	2.10
Creatinine	114.0662	POS	0.01004	0.00263	1.64
Proline Betaine	144.1018	POS	0.01004	0.00301	2.06
Carnitine	162.1124	POS	0.01013	0.00359	2.17
L-Histidine	154.0623	NEG	0.01347	0.00521	2.33
L-Methionine	148.0439	NEG	0.01825	0.00765	2.22
L-Glutamine	145.0618	NEG	0.01935	0.00874	2.45
L-Serine	104.0353	NEG	0.02129	0.01030	1.83
Guanidino butyric acid	146.0924	POS	0.02472	0.01276	1.90
L-Glutamate	146.0459	NEG	0.03642	0.02084	1.86
L-Tyrosine	182.0810	POS	0.03642	0.02115	1.87
L-Tryptophan	203.0828	NEG	0.04184	0.02565	1.81
L-Kynurenine	209.0921	POS	0.04405	0.02842	1.47
GABA	102.0561	NEG	0.04526	0.03212	1.84
L-Phenylalanine	164.0719	NEG	0.04526	0.03103	1.69
Ribose-phosphate (Pentose Phosphate)	229.0119	NEG	0.07132	0.05291	2.42
L-Threonine	120.0655	POS	0.14967	0.11588	1.40
L-Leucine	132.1019	POS	0.22593	0.18220	1.33
Methylhistidine	170.0924	POS	0.24036	0.20309	1.29
Taurine	126.0219	POS	0.24036	0.20934	1.40
Creatine	130.0623	NEG	0.39856	0.37284	1.70
Malate	133.0143	NEG	0.39856	0.36562	1.18
Glutamate 5-semialdehyde	132.0655	POS	0.70020	0.67761	0.93
Hypotaurine	110.0270	POS	0.87053	0.87053	1.04

Supplementary Table 4 Gender information of 98 health individuals obtained from a biobank (see Methods) that were analyzed using our FI-MS method

ID	Gender	ID	Gender
99	Male	148	Male
100	Male	149	Male
101	Male	150	Male
102	Male	151	Male
103	Male	152	Male
104	Male	153	Male
105	Male	154	Male
106	Male	155	Male
107	Male	156	Female
108	Male	157	Female
109	Male	158	Female
110	Male	159	Female
111	Male	160	Female
112	Male	161	Female
113	Male	162	Female
114	Male	163	Female
115	Male	164	Female
116	Male	165	Female
117	Male	166	Female
118	Male	167	Female
119	Male	168	Female
120	Male	169	Female
121	Male	170	Female
122	Male	171	Female
123	Male	172	Female
124	Male	173	Female
125	Male	174	Female
126	Male	175	Female
127	Male	176	Female
128	Male	177	Female
129	Male	178	Female
130	Male	179	Female
131	Male	180	Female
132	Male	181	Female
133	Male	182	Female
134	Male	183	Female
135	Male	184	Female
136	Male	185	Female
137	Male	186	Female
138	Male	187	Female
139	Male	188	Female
140	Male	189	Female
141	Male	190	Female
142	Male	191	Female
143	Male	192	Female
144	Male	193	Female
145	Male	194	Female
146	Male	195	Female
147	Male	196	Female

Supplementary Table 5 Mass accuracy in measurements performed with our optimized ranges FI-MS method on metabolite extracts from serum samples (using a Thermo Q Exactive mass spectrometer) based on a mix of internal standards.

Compound	Ion type	*m/z*	ppm error (99th percentile across 512 analysed samples)
Tryptophan (11C^{13})	$[M+H]^+$	216.1340	2.61
	$[M-H]^-$	214.1195	4.28
puromycin	$[M+H]^+$	472.2303	4.38
	$[M-H]^-$	470.2157	3.97
kiton red 620	$[M+H]^+$	581.1387	4.22
	$[M-Na]^-$	557.1422	4.13

Supplementary References

1. Wishart, D. S. *et al.* HMDB 4.0: the human metabolome database for 2018. *Nucleic Acids Res.* **46,** D608–D617 (2018).
2. Sud, M. *et al.* LMSD: LIPID MAPS structure database. *Nucleic Acids Res.* **35,** 527–532 (2007).
3. Dunn, W. B. *et al.* Molecular phenotyping of a UK population: defining the human serum metabolome. *Metabolomics* **11,** 9–26 (2014).
4. Frezza, C. *et al.* Metabolic profiling of hypoxic cells revealed a catabolic signature required for cell survival. *PLoS One* **6,** (2011).
