# Supplementary material for: Fast and sensitive flow-injection mass spectrometry metabolomics by analyzing sample-specific ion distributions
Source: Nat Commun. 2020 Jun 24;11:3186. doi: 10.1038/s41467-020-17026-6 (PMC7314751; doi:10.1038/s41467-020-17026-6)
Supplement: Supplementary file 3 — Description of Additional Supplementary Files [file 41467_2020_17026_MOESM3_ESM.docx]

File Name: Supplementary Data 1

Description: *m/z* features detected in serum samples with our optimized ranges FI-MS method (applied in both negative and positive ionization modes) and corresponding putative annotations.

File Name: Supplementary Data 2

Description: Inter-subject variability in serum metabolome abundances within a group of 98 healthy individuals based on measuremnets performed with our optimized ranges FI-MS method.

File Name: Supplementary Data 3

Description: *m/z* features detected in HeLa cells and media extracts with our optimized ranges FI-MS method (applied in both negative and positive ionization modes) and corresponding putative annotations.

File Name: Supplementary Data 4

Description: Pearson correlation between ion intensity measurements performed by our optimized ranges FI-MS method across 10 cell lines and LC-MS measurements.

File Name: Supplementary Data 5

Description: Annotation of m/z features identified with out optimized ranges FI-MS method via MS/MS.
